# The Geographical Distribution of Morbidity Caused By Chronic Obstructive Pulmonary Disease in Turkey: COPDTURKEY-2

**DOI:** 10.4274/balkanmedj.galenos.2020.2019.10.79

**Published:** 2020-04-10

**Authors:** Mustafa Hamidullah Türkkanı, Tarkan Özdemir, Hatice Kılıç, Nilgün Yılmaz Demirci, Çiğdem Özdilekcan, H. Canan Hasanoğlu, Orhan Koç, Can Öztürk

**Affiliations:** 1 Clinic of Chest Diseases, Sincan Dr. Nafiz Körfez State Hospital, Ankara, Turkey; 2 Clinic of Chest Diseases, University of Health Sciences, Dr. Abdurrahman Yurtaslan Ankara Oncology Research and Training Hospital, Ankara, Turkey; 3 Department of Chest Diseases, Yıldırım Beyazıt University School of Medicine, Ankara, Turkey; 4 Department of Chest Diseases, Gazi University School of Medicine, Ankara, Turkey; 5 Deputy President, Social Security Institution, Ankara, Turkey

**Keywords:** Chronic obstructive pulmonary disease, prevalence, morbidity, Turkey

## Abstract

**Background:**

Chronic obstructive pulmonary disease (COPD) is one of the most prevalent causes for morbidity and mortality, and it creates a cumulative economic and social burden.

**Aims:**

To determine the distribution of the prevalence of patients in Turkey who were diagnosed with COPD and their morbidity rates, according to the regions and cities they belong to. Moreover, the study contributes to the prevention and cure services of COPD that should be planned in the future.

**Study Design:**

A retrospective cohort.

**Methods:**

The database of the Social Security Institution from 2016 has been scanned. All the data with prescription registration, with the code ICD-10, J44.0-J44.9, which were aimed for diagnosing and/or cure, have been evaluated with a retrospective cohort.

**Results:**

In 2016, 955,369 patients who were admitted as outpatients to the hospitals were diagnosed with COPD. The average number of annual COPD cases that were admitted was 2.09. Twenty percent (20%) of the outpatient applications were via emergency room. The rate of hospitalization among the applicants was 17.75%, with a total of 1,994,325. The average annual number of hospitalizations of men was higher than that of women. The average number of hospitalization days was 6.52. The region with the highest prevalence of outpatient admission and hospitalization was the Black Sea Region.

**Conclusion:**

The high rate of hospitalization was considered to be the outcome of the insufficient “outpatient” management.

Even though chronic obstructive pulmonary disease (COPD) is a preventable and curable disease, it is still a worldwide public health problem. COPD is one of the most prevalent causes for morbidity and mortality, and it creates a cumulative economic and social burden (1,2). The high rates of smoking, air pollution, and aging of the population all contribute to the COPD being a great issue not only in present but also in the future (3). Awareness and financial aids directed towards pulmonary diseases are less, in general, compared to other global morbidity and mortality causes (3). Diseases which cause comparable morbidity and mortality and result in high costs are not as neglected as COPD by healthcare service providers (4).

Morbidity measures in COPD include traditional outpatient polyclinic admissions, emergency room (ER) service admissions, and hospitalizations (5). These data cannot be obtained often, and the confidentiality of the obtained data is almost controversial, compared to mortality rates (4). The main reason for this is the differences and the comorbidities between the healthcare systems of the countries and discrepancies about diagnostic and treatment modalities (6). Moreover, the measures used in morbidity evaluation can be affected by external factors, such as hospital bed capacity, referral chain between healthcare institutions, and social security institutions’ rules. Despite all of these factors, morbidity data are still effective enough to estimate the need for healthcare services (7,8).

In Turkey, where smoking habits and household air pollution are prevalent, enough research was not performed on the morbidity caused by COPD (9). In the study about the national burden of the disease, which was performed in 2004, COPD was found to be the 8th on the list of top ten illnesses that cause national burden, with a rate of 2.8% (10).

This study aims to show the data about the morbidity caused by COPD in Turkey, in terms of the geographical distribution of the disease. The study contributes to highlighting the scientific researches, especially the epidemiologic ones, and the health policies concerned with COPD.

## MATERIALS AND METHODS

### Data source

This study was a population-based, observational, descriptive, surveillance study. In order to obtain data of this study, an official application was submitted to the Social Security Institution (SSI) which is a pay-back institution and involves 98.6% of the public. Data are obtained from an information system (named MEDULA), which is also used by SSI and proceeds demands for all the health insurance companies in Turkey. Data about hospitalized patients and outpatient admissions, obtained from MEDULA, was involved with 875 public hospitals, 70 university hospitals, 565 private hospitals, and 25,000 pharmacies. MEDULA contained data about outpatient admissions medical data entered by doctors in case of hospitalizations, patients’ demographic information, and observed clinical improvements and vital clinical results.

### Population of the study

The data on the MEDULA system, which was collected between January and December 2016 with diagnostic and treatment purposes, was evaluated retrospectively within the data of patients diagnosed with COPD, over the age of 15, and with the codes ICD-10, J44.0-J44.9. The data was collected from different hospitals. Demographic data, such as the quantity of patients, gender distribution, and average age gap, as well as morbidity data, such as polyclinic admissions number, ER service admissions number, and hospitalized patients’ number, are examined in MEDULA, by using the program Tool for Oracle Application Developers (TOAD) 9.6.0.27. This study’s cohort was made up of extracted, anonymized patients' data.

Ethics committee approval was received for this study from the local ethics committee (date: 14.11.2018, no: 2018-11/136).

### Statistical analysis

SAS Enterprise Guide 5.1 statistical program (SAS Institute Inc. Cary, North Carolina) was used for statistical evaluation. For the calculations of outpatient admissions and hospitalizations, the population of Turkey over the age of 15 was included (11).

## RESULTS

In the year of 2016, there have been 955,369 COPD patients [64.9% (n=619,932) being male patients] and 1,994,325 admissions to the hospitals. The average age of patients was 65.79±13.63. The average number of admissions to the hospitals per patient was 2.09. The average admissions number of males was higher than that of the females (2.27 vs. 1.74, respectively) (Table 1). In the same year of the 218,478 patients, 64.1% (n=140,150) were the male patients those have been hospitalized. The average age gap in hospitalized patients was 71.62±11.95. The average frequency of hospitalization per patient was 1.62. The average age of females who were hospitalized was higher than that of the males (73.04±12.39 vs. 70.76±11.61, respectively). The average frequency of hospitalization of males was higher than that of females (1.71 vs. 1.54) (Table 1).

In 2016, 20% (n=406,982) of COPD outpatients were admitted to the ER, and the remaining 80% (n=1,587,343) were admitted to polyclinics. The average number of days of hospitalization was 6.52. The percentage of ambulatory patients who were indicated to be hospitalized was 17.75% (Table 2).

The region which has the highest percentage of outpatient admissions and prevalence of hospitalization of COPD patients was the Black Sea Region (1.01% and 4.71%, respectively), while the lowest percentage of prevalence of hospitalization was in the Marmara Region (0.34%) and the lowest percentage of outpatient admissions was in the Southeast Anatolian Region (1.44%) (Table 3).

**Figure 1:**
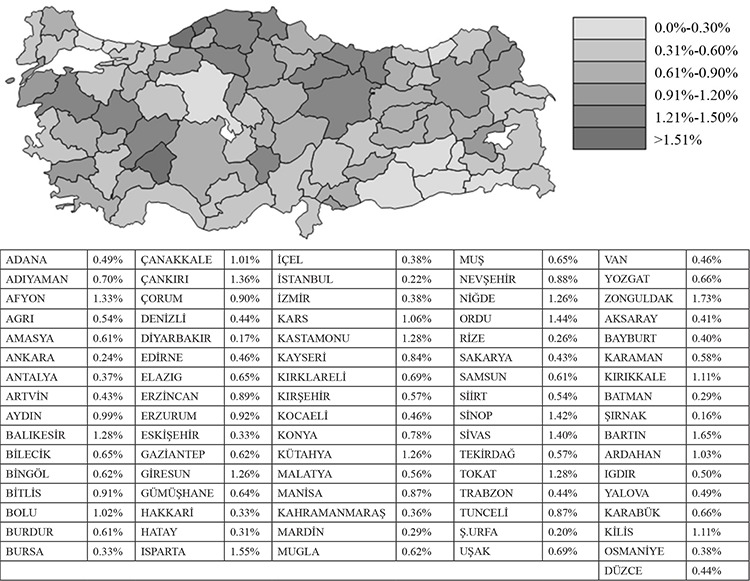
The distribution of prevalence of hospitalization of patients with chronic obstructive pulmonary diseases (COPD) in Turkey, according to the provinces, 2016

When the prevalence of hospitalization, in terms of the provinces, was evaluated, it was seen that the distribution was between 0.16 and 1.73%, and the highest prevalence was in Zonguldak, and the lowest was in Şırnak (Figure 1).

**Figure 2:**
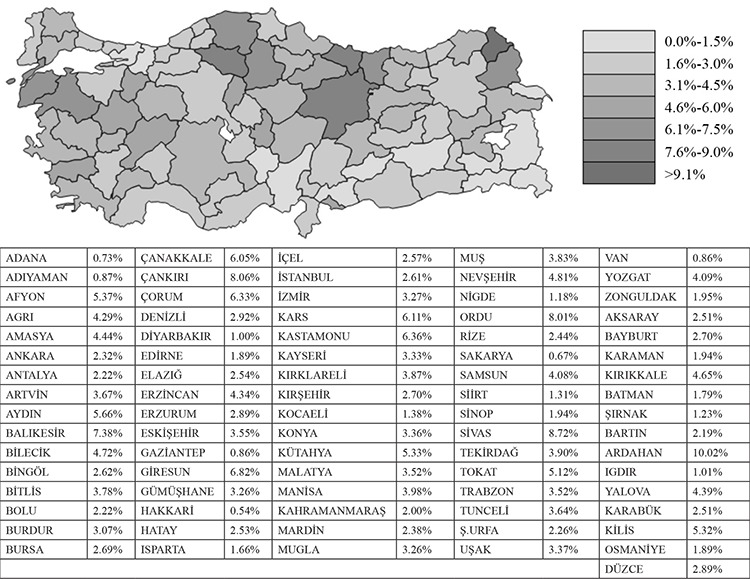
The distribution of prevalence of outpatient admissions of patients with chronic obstructive pulmonary diseases (COPD) in Turkey, according to the provinces, 2016

When the prevalence of outpatient admissions, in terms of the provinces, was evaluated, it was observed that the highest prevalence was in Ardahan (10.02%), and the lowest was in Hakkari (0.54%) (Figure 2).

## DISCUSSION

This study is the first one that shows the morbidity results of COPD in Turkey using the national database. COPD is usually known to affect males more than females and the elderly people. In Greece, in 2012, there have been studies evaluating the models of writing prescriptions for COPD outpatients, using the data of the largest social security fund. According to this study, it has been determined that, in 174,357 COPD patients, the average age was 69.3±14.8 and 52% of them were males (12). In Sweden, in a study between 2009 and 2010, using the data of the National Health Institution Center of Epidemiology and the national database, it has been determined that there were 88,548 patients who have been diagnosed with COPD and chronic bronchitis. Their average age was 72.1±10.8, and 53.6% of the study group were females (13). Overall, morbidity increases with age in COPD patients (14).

According to our study, 64.9% of the outpatient admissions and 64.1% of the hospitalized patients were males. Even though the mean ages of outpatient admissions among males and females were similar, males were hospitalized at younger ages compared to the females. Thus, as an indication of morbidity, males have higher rates than women in terms of outpatient admissions and hospitalization. In males, a higher rate of exposure to tobacco creates great symptom burdens as well as comorbidities (15). Moreover, in Turkey, while in 2000 the percentage of the population over the age of 65 was 5.7%, in 2016, this number has been exceeded by 8.3% (16). The increase in elderly population and usage of tobacco as well as other risk factors cause a rise in the risk of contracting COPD.

Most of the information about COPD morbidity comes from high-income countries. In the USA, it was found that in 2000, COPD patients diagnosed by doctors had 8 million doctor offices and hospital polyclinic applications, 1.5 million emergency department applications, and 726,000 hospitalizations (17). In 2010, 10,291,000 doctors’ offices applications, 1,468,000 emergency department applications, and 699,000 hospitalizations were recorded (18).

In most areas in Turkey, there have been 469,718,440 outpatient admissions and 13,452,686 hospitalizations with 54,150,918 days of hospitalization (15). In light of this data, outpatient admissions resulting in the diagnosis of COPD are equivalent to 0.4% of the total outpatient admissions, 2.6% of the hospitalizations, and 4.3% of the total number of days of hospitalization. Even though the rate of hospitalization in Turkey is 2.6%, COPD’s rate of hospitalization is 17.75%, which can be considered to be a high rate of hospitalization. Out of 5 outpatient admissions, only one patient is treated by being hospitalized, which indicates that the management of the disease, the implementation of the pulmonary rehabilitation, and home healthcare which reduces the morbidity are insufficient.

In the nationwide study of hospital admissions covering Switzerland between 2002 and 2010, 2.6% of the hospitalized cases were diagnosed with COPD (19). COPD-related hospitalizations in the United States accounted for about 3.31% of all hospitalizations in 2002, up from 3.43% in 2010 (20).

The number of days of hospitalization is considered crucial morbidity data which shows the burden of the disease. Without the discrimination of the disease, in 2016, in Turkey, the average number of days of hospitalization was 4, while in the European Union 28, it was 7.9 days (21). While the average number of days of hospitalization was 5.3 days in cardiovascular diseases, in pulmonary diseases, this number was 5.6 days (15). In the USA, the average length of hospital stay in COPD patients decreased from 6.4 days in 2002 to 6.0 days in 2010 (20). In a study in London, England, in all the COPD patients, the average number of days of hospitalization was 8.0 in 2006. And in 2010, this number decreased to become 7.2 (22). In Blackpool, England, in a study that includes the years 2005 to 2010, the average number of days of hospitalization has been determined as 9.8 days (23). In New Zealand, in a study, which was carried out between 2008 and 2013, in 61.516 hospitalizations caused by COPD, it was observed that the average number of days of hospitalization has decreased from 5.09 to 4.37 days (24). In a study performed in Rome, Italy, it was determined that the average number of days of hospitalization is 12.25 days in 2010, 11.63 days in 2011, and 11.91 days in 2012 (25). According to our study, in COPD patients, the average number of days of hospitalization is found to be 6.52. This finding was evaluated as a reasonable period of time for the follow-up and treatment of the patient. Similar to hospitalizing the patients, it is thought that coordinating with “Home Health Care Service Units” is essential and it can reduce the period of time spent in the hospital and help to plan the discharge from the hospital earlier.

The region which has the highest percentage of outpatient admissions and prevalence of hospitalization in COPD patients is the Black Sea Region. According to the data of 2016, while the rate of the population in rural areas is 12.1% around Turkey, in the Western Black Sea Region, this rate is 33.3%, and in Eastern Black Sea Region, it is 31.7% (15). The rate of the increase in population, irregular population distribution, and geographical factors in rural areas are thought to be the reasons that cause the difficulty in reaching the hospital. They are also the reasons behind the preference of the patients being hospitalized, rather than being outpatients. Thus, in Australia, in many studies that were done in order to evaluate the difference in hospitalization rates between the rural populations and city populations, it was found that in the rural areas, there were less hospitalized COPD patients (26). It is thought that the reason why the diagnosis frequency of COPD is high is because of the Black Sea Region has the highest rate of the population over the age of 65, which is 12.8% (15). This region has a high rate of precipitation and humidity and has a large area of forestation that affects the number of pollens, and we recommend carrying out further studies to investigate this issue. Humidity and high temperatures are directly related to the worsening of symptoms in COPD patients whereas high humidity and low temperatures are shown to increase the risk of developing COPD (27).

The lowest percentage of prevalence of hospitalization is in the Marmara Region (0.34%). Even though the most populated region in Turkey is Marmara (28), the hospital bed number per 10,000 people is relatively lower than most of the regions (15). Therefore, we believe that one of the reasons for the low prevalence of hospitalization in Marmara is because of the insufficient number of beds.

The lowest percentage of outpatient admissions is in the Southeast Anatolian Region (1.44%). The lowest percentage of a population over the age of 65 is in the Southeastern Anatolian region, with 4.7% (15). This value is thought to be the reason for the fall in the values of COPD prevalence. Average temperatures are related to hospitalization numbers independently and closely (29). In the Southeastern Anatolia Region, we believe that factors such as the warm climate and effective air conditioning in indoor areas reduce the risks of infections; also, the sparse vegetation cover and the short lifespan of the plants contribute to the control of the disease.

When the distribution of the outpatient admissions is evaluated based on their cities, the prevalence is between 0.54 and 10.02%, while the prevalence of hospitalization values is between 0.16 and 1.73%. We believe that the difference between the values is caused by the average number of patients per physician, hospital bed number, health literacy, sociocultural factors, the importance given to the preventive healthcare services, the consistency and prevalence of the healthcare services at home, and the differences between reaching the healthcare services. In the guidelines, it is mentioned that the medical treatments of COPD should have a symptomatic approach and the essential aim is to reduce the symptoms of the disease and the future risks of the disease. Also, it is mentioned that with proper medical treatment, the frequency of exacerbations and the severity of illness will be reduced; health situation and exercise tolerance will progress. A good COPD treatment is composed of pharmacological and nonpharmacological approaches. Education of the patients, reducing the environmental risk factors or removing them, quitting smoking, pulmonary rehabilitation, and physical activity are few of the nonpharmacological approaches that can be carried out (5,6,30,31,32,33). The prevalence values based on cities and regions in our study show that the treatment of COPD, which aims to treat the symptomatic aspects, is mainly done pharmacologically, without considering the use of the nonpharmacological approaches. In light of these findings, ministries should change their policies to be more directed towards the nonpharmacological treatments.

Our study is designed on the basis of only hospital admissions of COPD patients. The database of admissions for the primary based healthcare services (family practice) is not included in the study and this can be considered as a limitation in this study.

In addition to the overall morbidity data, regional and provincial differences were determined in our study. It was thought that this data would be a guide for the analytical work. Morbidity being high in the males, which is a higher risk group, shows that the fight against tobacco products should be continued with the utmost effort and health literacy should be a priority. The high rates of hospitalization show that the outpatient admissions should be increased and “Home Healthcare Services” should play a more active role. The difference in the distribution in different geographical regions should be further investigated through analytical studies.

## Figures and Tables

**Table 1:**
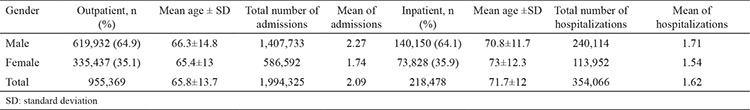
The demographic features of outpatient and inpatient chronic obstructive pulmonary diseases patients and morbidity data, 2016

**Table 2:**
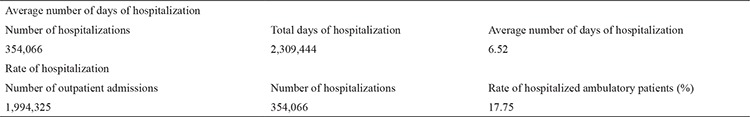
The average number of days of hospitalization and the percentage of hospitalization in chronic obstructive pulmonary diseases patients, 2016

**Table 3:**
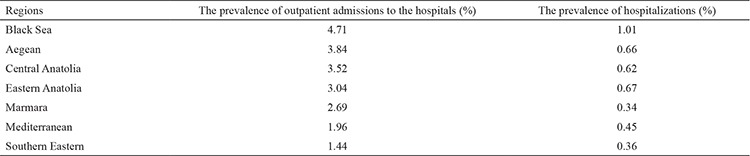
The geographical distribution of outpatient admissions and prevalence of hospitalization of chronic obstructive pulmonary diseases patients in Turkey, 2016
